# Effects of Dietary Cereal and Protein Source on Fiber Digestibility, Composition, and Metabolic Activity of the Intestinal Microbiota in Weaner Piglets

**DOI:** 10.3390/ani12010109

**Published:** 2022-01-04

**Authors:** Carola Ellner, Anna G. Wessels, Jürgen Zentek

**Affiliations:** Department of Veterinary Medicine, Institute of Animal Nutrition, Freie Universität Berlin, 14195 Berlin, Germany; anna.wessels@fu-berlin.de (A.G.W.); juergen.zentek@fu-berlin.de (J.Z.)

**Keywords:** cereal, dietary fiber, microbiome, nutrient digestibility, pigs, protein source, rapeseed, rye, soybean, wheat

## Abstract

**Simple Summary:**

Rye and rapeseed meal can be alternative feed components for weaner piglets instead of wheat and soybean meal. Both components can help to meet current challenges in pig nutrition, such as increasingly dry weather conditions and the high amount of imported soybean. Since they contain more and differently composed fiber, effects on digestive physiology and intestinal microbiota might help to maintain gut health and prevent post-weaning diarrhea. This study shows that despite a similar composition of the large intestinal microbiota, the higher amount and solubility of complex carbohydrates from rye lead to a higher fermentative activity compared to wheat, which is considered a beneficial effect. The high amount of insoluble dietary fiber in rapeseed-based diets lowered bacterial metabolic activity and caused a shift toward insoluble fiber degrading bacteria.

**Abstract:**

This study aimed to investigate the effect of fiber-rich rye and rapeseed meal (RSM) compared to wheat and soybean meal (SBM) on fiber digestibility and the composition and metabolic activity of intestinal microbiota. At weaning, 88 piglets were allocated to four feeding groups: wheat/SBM, wheat/RSM, rye/SBM, and rye/RSM. Dietary inclusion level was 48% for rye and wheat, 25% for SBM, and 30% for RSM. Piglets were euthanized after 33 days for collection of digesta and feces. Samples were analyzed for dry matter and non-starch-polysaccharide (NSP) digestibility, bacterial metabolites, and relative abundance of microbiota. Rye-based diets had higher concentrations of soluble NSP than wheat-based diets. RSM-diets were higher in insoluble NSP compared to SBM. Rye-fed piglets showed a higher colonic and fecal digestibility of NSP (*p* < 0.001, *p* = 0.001, respectively). RSM-fed piglets showed a lower colonic and fecal digestibility of NSP than SBM-fed piglets (*p* < 0.001). Rye increased jejunal and colonic concentration of short-chain fatty acids (SCFA) compared to wheat (*p* < 0.001, *p* = 0.016, respectively). RSM-fed pigs showed a lower jejunal concentration of SCFA (*p* = 0.001) than SBM-fed pigs. Relative abundance of *Firmicutes* was higher (*p* = 0.039) and of *Proteobacteria* lower (*p* = 0.002) in rye-fed pigs compared to wheat. RSM reduced *Firmicutes* and increased *Actinobacteria* (jejunum, colon, feces: *p* < 0.050), jejunal *Proteobacteria* (*p* = 0.019) and colonic *Bacteroidetes* (*p* = 0.014). Despite a similar composition of the colonic microbiota, the higher amount and solubility of NSP from rye resulted in an increased fermentative activity compared to wheat. The high amount of insoluble dietary fiber in RSM-based diets reduced bacterial metabolic activity and caused a shift toward insoluble fiber degrading bacteria. Further research should focus on host–microbiota interaction to improve feeding concepts with a targeted use of dietary fiber.

## 1. Introduction

An important approach to stabilize gut health of weaner pigs is the optimization of intestinal microbial colonization in the sense of intestinal eubiosis and beneficial bacterial metabolic activity [1]. Here, the inclusion of dietary fiber is a promising strategy. Dietary fiber influences digestion and fermentation processes and many sources of dietary fiber were shown to increase the potentially beneficial microbiota, to reduce pathogens, and to improve intestinal barrier function [2,3,4,5]. Non-starch-polysaccharides (NSP) are the major component of dietary fiber. They are not digestible in the small intestine but are fermented in the upper and lower intestinal tract by the resident microbiota [6]. Fermentation products of intestinal bacteria can have different impact on the microbial community and on the host. Proteolytic bacterial activities produce potentially harmful metabolites such as ammonia and other degradation products [7]. Beneficial metabolites are short-chain fatty acids (SCFA) and lactic acid. They are products of substrate fermentation and may hamper the growth of pathogens and provide energy for beneficial bacteria [8]. Acetic, propionic, and butyric acid are the predominant forms of SCFA. After absorption, they serve as an energy substrate for the pig [9]. Moreover, butyric acid is the main energy source of the colonic epithelial cells [10]. Most intestinal bacteria preferably ferment carbohydrates including NSP, which implies that diets containing high amounts of complex structured NSP might improve gut health by promoting growth of fermentative bacteria and providing beneficial metabolites to the host. Dietary fiber may also influence the integrity of the epithelial mucus layer and on mucus production [11]. *N*-acetylneuraminic acid is a sialic acid present in mucin molecules and can be considered a marker for mucus [12], but data on its intestinal concentration in pigs fed different fiber sources are scarce.

Rye and rapeseed meal (RSM) are fiber-rich feed components and interesting alternatives to wheat and soybean meal (SBM). Rye is not used much in pig nutrition due to the idea of low palatability, its high content of fiber and its susceptibility to the infection with *Claviceps purpurea* [13]. However, recent studies investigating the feeding of rye instead of wheat at dietary inclusion up to 69% showed no reduction of feed intake and growth performance [14,15]. This might be related to the use SCFA from large intestinal fiber fermentation as an energy substrate [16]. Moreover, increasing pollen fertility and selection of resistance genes lowered the risk of ergot infections in rye [17]. Generally, wheat and rye have a similar concentration of carbohydrates, but rye has a higher concentration of total dietary fiber (TDF) and fermentable fractions [16]. The content of total NSP, soluble NSP (sNSP), arabinoxylans (AX) and β-glucans is higher in rye compared to wheat [18,19]. Thus, rye increases intestinal butyrate production compared to wheat [20,21] and helps to prevent *Salmonella* infection in weaner pigs [15]. Moreover, rye might be effective against enterotoxigenic *E. coli* (ETEC) colonization [5] and promote the growth of *Bifidobacterium* and *Prevotella* spp., both important lactic acid-producing bacteria. Lactic acid is considered to inhibit growth of pathogens [22]. A high abundance of proteolytic *Clostridium* can impair gut health and might be reduced by increasing dietary AX and β-glucans [23]. Consequently, replacing wheat by rye might induce a favourable shift in the large intestinal microbiome by providing more readily fermentable substrate and thereby promoting the production of useful metabolites.

Next to SBM, RSM is the second most important protein meal in pig nutrition in Europe. RSM and SBM contain similar amounts of NSP, but RSM has less soluble NSP and more lignin, total, and soluble AX [18]. Insoluble fiber helps to maintain normal gut function but might decrease feed intake and nutrient digestibility [9]. Very little research has been done on the fermentability of RSM carbohydrates. However, some studies showed that RSM might increase relative abundance of SCFA producing genera in the hindgut and decrease major proteolytic bacteria compared to SBM [24,25].

Providing non-digestible fiber substrates using rye and RSM in piglet diets could support gut health. Knowledge of the effects of feeding rye in combination with RSM is scarce. For the present study, we hypothesized that rye and RSM would increase microbial fiber fermentation and SCFA production in the large intestine compared to wheat and SBM and cause a shift in the microbial community toward potentially beneficial bacteria. The aim of this study was to investigate the effect of diets containing either rye or wheat as a cereal combined with either RSM or SBM as a protein meal on fiber digestibility, on the mucus marker *N*-acetylneuraminic acid, as well as on composition and metabolic activity of intestinal microbiota.

## 2. Materials and Methods

### 2.1. Animals and Diets

The animal study was described in detail previously [14]. In brief, 88 piglets (German Landrace, bred at the Institute of Animal Nutrition, Freie Universität Berlin, 8.3 ± 1.1 kg body weight) were weaned at day 28 of life and randomly allocated to four feeding groups in a 2 × 2 factorial design with wheat/SBM (W-SBM), wheat/RSM (W-RSM), rye/SBM (R-SBM), and rye/RSM (R-RSM). Piglets were housed with two animals per pen and 11 pens per treatment. The pen was used as statistical unit (*n* = 11). The health status was monitored daily by controlling general condition, feed intake, and fecal consistency of the piglets. Water and feed were supplied ad libitum during the experimental period of 33 days. The diets were formulated to meet or exceed the recommendations for piglet nutrition [26] and calculated to be isonitrogenous. Wheat (*Triticum aestivum*) or hybrid rye (*Secale cereale*) were included as cereals at 48 and 25% of SBM or 30% of RSM as protein rich ingredient. The average concentration of crude protein in the complete diet was 220 g/kg. Crude fiber varied among the groups with 22 g/kg in W-SBM, 52 g/kg in W-RSM, 18 g/kg in R-SBM, and 48 g/kg in R-RSM. The exact feed formulation, results of the nutrient analysis, and digestibility determinations are available elsewhere [14].

### 2.2. Sampling

One piglet per pen was dissected after 33 days of trial duration. Pigs were chosen to achieve balanced numbers of males and females in each treatment group. After anaesthesia by 20 mg of ketamine hydrochloride (Ursotamin, 10%; Serumwerk Bernburg AG, Bernburg, Germany) and 2 mg of xylazine (Xylazin, 2%, Serumwerk Bernburg AG, Bernburg, Germany) per kg body weight, pigs were euthanized with an intracardial injection of tetracaine hydrochloride, mebezonium iodide, and embutramide (T61; Intervet, Unterschleißheim, Germany). The gastrointestinal tract was removed and digesta samples were collected from jejunum, ileum, colon ascendens, and rectum. From each intestinal segment, one subset of digesta samples was stored at −20 °C for analysis of dry matter (DM), fiber and *N*-acetylneuraminic acid. Another subset was snap frozen with liquid nitrogen and stored at −80 °C for analysis of bacterial metabolites and 16S rRNA sequencing.

### 2.3. Determination of Digesta Content and Apparent Digestibility of DM and NSP

Digesta samples from jejunum, colon ascendens, and feces were dried in an oven at 103 °C overnight to determine the DM content. Colonic and fecal samples were lyophilized and total and insoluble NSP content was measured as described for NSP in the diets [14]. To determine apparent digestibility of DM and NSP, titanium dioxide content was determined as described before [27] in lyophilized and grinded (0.5 mm particle size) digesta, feces, and in the diets. Apparent digestibility of DM and NSP was calculated as follows [28]:

Digestibility (%) = 100 − (TiO2 in feed (%))/(TiO2 in digesta/feces (%)) × (Nutrient in digesta/feces (%))/(Nutrient in feed (%)) × 100

Apparent digestibility determined in feces was further considered as apparent total tract digestibility (ATTD).

### 2.4. Analysis of N-Acetylneuraminic Acid as Marker of Intestinal Mucus Production

Lyophilized and grinded samples of ileal digesta were hydrolyzed with acetic acid (2 mol/L). After centrifugation, the supernatant was analyzed for *N*-acetylneuraminic acid via HPIC with an amperometrically pulsed detector cell (Thermo Fisher Scientific, Waltham, MA, USA) using a commercial standard of *N*-acetylneuraminic acid as reference substance (Sigma–Aldrich, Taufkirchen, Germany).

### 2.5. Analysis of Bacterial Metabolites

In jejunal and colonic digesta samples and feces, concentration of SCFA, d- and l-lactate, and ammonia was analyzed as described before [29]. Briefly, SCFA were determined via gas chromatography (Agilent Technologies 6890N, autosampler G2614A, and injection tower G2613A; Network GC Systems, Böblingen, Germany) using caproic acid as an internal standard. d- and l-lactate were measured by HPLC (Agilent 1100; Agilent Technologies, Böblingen, Germany). Ammonia was analyzed colorimetrically using the Berthelot reaction in microtitration plates at 620 nm in a Tecan Sunrise microplate reader (Tecan Austria GmbH, Grödig, Austria).

### 2.6. DNA Extraction and 16S rRNA Sequencing

To extract total DNA from 0.25 g of jejunal and colonic digesta and feces, a commercial extraction kit (QIAamp PowerFaecal Pro DNA Kit, Qiagen, Hilden, Germany) was used in accordance with the manufacturer’s instructions with an additional lysis step at 65 °C. Homogenization was carried out using FastPrep-24^TM^ 5G (M.P. Biomedicals LLC, Santa Ana, CA, USA) at 6 m/s for 10 min (4 times 5 × 30 s and 15 s pause time). DNA extracts were stored at −30 °C until further analysis. Extracts were subjected to amplicon sequencing using an Illumina NextSeq500 sequencer (LGC, Berlin, Germany) with two 150–base pair reads. After demultiplexing, BBMerge tool [30] was used for combining paired reads. Resulting 16S rDNA sequences were analyzed using QIIME2 pipeline [31] and the SILVA SSU database [32]. Quality control and determination of sequence counts were performed using the DADA2 [33]. Further details were described previously [34]. Indices of bacterial diversity (Richness, Shannon index, and Evenness) were calculated from ASV level data. Principal component analysis of 16S rRNA data was carried out using the online software ClustVis [35].

### 2.7. Statistical Analysis

Statistical analyses were carried out in SPSS 26.0 (IBM, Chicago, IL, USA). The distribution of data was tested by Kolmogorov–Smirnov test. Normally distributed data were analyzed by 2-factorial ANOVA with cereal (CER) and protein meal (PM) as fixed factors. Group differences were assessed by post hoc Tukey test. *p*-values below 0.05 were considered significant. Pearson‘s correlation was analyzed between colonic *Bacteroidetes* and average daily gain (ADG) and between jejunal *Proteobacteria* and apparent ileal digestibility (AID) of crude protein and total amino acids.

## 3. Results

Results of growth performance and fecal score were described in detail previously [14]. In brief, average daily gain, average daily feed intake, feed conversion ratio and final body weight were not influenced by the feeding of rye compared to wheat. RSM in comparison to SBM reduced average daily gain and average daily feed intake in the overall trial period. Fecal score was within the physiological range throughout the trial.

### 3.1. NSP Concentration in Diets

The four experimental diets contained more iNSP (insoluble non-starch-polysaccharides, 76.9–93.0 g/kg) than sNSP (12.8–34.6 g/kg) with glucose (27.0–48.2 g/kg), xylose (24.2–30.6 g/kg), and arabinose (18.2–30.5 g/kg) being the predominant sugars in the NSP fractions (Table 1). The two rye-based diets had higher concentrations of soluble dietary fiber (SDF) and sNSP than the wheat-based diets (47 and 118% more, respectively). RSM-diets were higher in iNSP and the A/X-ratio was higher compared to SBM-diets (15 and 35% more, respectively).

### 3.2. Apparent Digestibility of DM and NSP

Fecal DM digestibility was lower in rye-fed piglets than pigs receiving wheat-based diets (*p* = 0.035, Table 2). DM digestibility was lower in RSM-pigs than in SBM-pigs in the jejunum, colon and feces (each *p* < 0.001).

Colonic NSP digestibility (70.7–88.2%) was higher than fecal NSP digestibility (59.5–74.8%) across the four feeding groups. Both sites showed a higher digestibility of soluble than of insoluble NSP and AX. Rye-fed piglets showed a higher colonic and fecal digestibility of NSP, sNSP, glucose and soluble AX (colon: each *p* < 0.001; feces: *p* = 0.001, *p* < 0.001, *p* < 0.001, *p* = 0.005, respectively). Compared to SBM, RSM-fed piglets showed a lower colonic and fecal digestibility of NSP and iNSP (each *p* < 0.001). Fecal digestibility of iNSP was highest in R-SBM-fed pigs and lowest in R-RSM-fed pigs (*p* < 0.001). Fecal digestibility of insoluble AX was highest in W-RSM and lowest in R-RSM-fed piglets (*p* = 0.009).

### 3.3. Concentration of N-Acetylneuraminic Acid in the Ileum Digesta

Ileal digesta concentration of *N*-acetylneuraminic acid and ratio of *N*-acetylneuraminic acid to titanium dioxide were measured to estimate mucus production in the small intestine. Nor concentrations neither the ratio were affected by the dietary treatments (*p* > 0.05, Table 3).

### 3.4. Bacterial Metabolites

Concentration of SCFA was highest in colonic digesta, followed by feces and jejunal digesta (Table 4). Acetic acid was the predominant fraction of SCFA followed by butyric acid in jejunal digesta and by propionic acid in colonic digesta and feces. In comparison to wheat, rye increased concentration of SCFA (*p* < 0.001), acetic acid (*p* < 0.001), and propionic acid (*p* = 0.024) in the jejunum and of SCFA (*p* = 0.016), acetic acid (*p* = 0.014), propionic acid (*p* = 0.034), and i-butyric acid (*p* = 0.035) in the colon. RSM-fed pigs showed a lower concentration of SCFA (*p* = 0.001), acetic acid (*p* = 0.002), and n-butyric acid (*p* = 0.010) in the jejunum and a lower colonic concentration of acetic acid (*p* = 0.049), i-butyric acid (*p* = 0.001), n-butyric acid (*p* = 0.041), and i-valeric acid (*p* = 0.002) than SBM-pigs.

In caudal direction, ammonia increased, and l-lactate decreased. Ammonia, d-, and l-lactate were not affected by the dietary treatments (*p* > 0.05).

### 3.5. Microbial Diversity Indices

Microbial richness was reduced in jejunal digesta of piglets fed rye compared to wheat (*p* = 0.047; Table 5). Shannon index and evenness were not affected (*p* > 0.05).

### 3.6. Relative Abundance of Bacterial Phyla, Order, and Genera

Most abundant phyla in jejunum, colon, and feces were *Firmicutes* and *Bacteroidetes* (Table 6). In rye-fed pigs, jejunal *Firmicutes* were higher and *Proteobacteria* lower than in wheat-fed pigs (*p* = 0.039, *p* = 0.002, respectively). Compared to SBM, RSM reduced *Firmicutes*, and increased *Actinobacteria* at the three sampling sites (*p* < 0.050). RSM increased *Proteobacteria* in the jejunum and *Bacteroidetes* in the colon (*p* = 0.019, *p* = 0.014, respectively). Correlation (r) between the relative abundance of jejunal *Proteobacteria* and AID of crude protein was 0.119 (*p* = 0.484) and with AID of total amino acids it was 0.097 (*p* = 0.605). Correlation (r) between colonic *Bacteroidetes* and ADG of days 0–33 was −0.106 (*p* = 0.498) and of days 28–33 −0.302 (*p* = 0.049). In the jejunum, W-RSM-fed pigs showed a lower relative abundance of *Firmicutes* compared to the other three groups (*p* = 0.002).

At the genus level, rye increased relative abundance of *Clostridium sensu stricto 1* in the jejunum (*p* = 0.005) and *Terrisporobacter* in the jejunum and feces (*p* = 0.018, *p* = 0.004, respectively). Compared to SBM, RSM increased *Bifidobacterium* spp. in the jejunum (*p* = 0.046) and the genera *Prevotella 9, Blautia,* and *Syntrophococcus* in feces (*p* = 0.022, *p* = 0.039, *p* = 0.024, respectively). RSM decreased *Clostridium sensu stricto 1* in the three sampling sites (*p* < 0.05), *Terrisporobacter* in jejunum and feces (*p* = 0.033, *p* = 0.004, respectively), and unknown *Ruminococcaceae* and *Christensenellaceae R-7* group in feces (*p* = 0.042, *p* = 0.031, respectively). Results at the order level are displayed in the Supplementary Materials.

**Figure 1:**
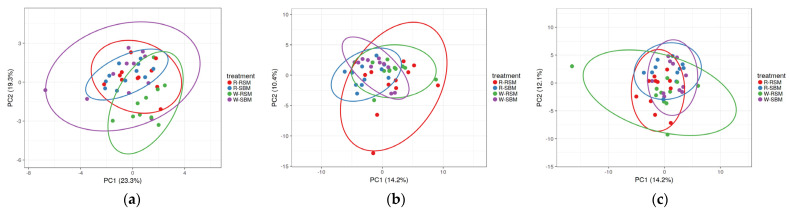
PCA showing the effect of the experimental diets on the relative abundance (%) of bacterial genera in digesta of jejunum (**a**), colon ascendens (**b**), and feces (**c**) in weaned piglets; R-SBM, rye/soybean meal; R-RSM, rye/rapeseed meal; W-SBM, wheat/soybean meal; W-RSM, wheat/rapeseed meal. Principal component analysis was performed with ClustVis.

Principal component analysis showed higher variabilities of the relative abundance of bacterial genera in jejunal digesta (≤23.3%) than in colonic digesta and feces (≤14.2%; Figure 1). Clusters of the experimental diets were overlapping to a high extent.

## 4. Discussion

### 4.1. Fiber Composition of the Experimental Diets

The use of fiber-rich feed components might help to stabilize the intestinal milieu during the post-weaning period. As rye contains more SDF and sNSP [18,36] the rye-based diets used in the current study were characterized by higher concentrations of soluble fiber fractions than the wheat-based diets. Similarly, the high concentrations of iNSP in RSM [18] resulted in a higher concentration of iNSP in the RSM-based diets compared to the SBM-based ones.

AX are the predominant fraction of cereal NSP and consist of a xylan backbone substituted by arabinose to varying degrees [18]. The degree of substitution can be described by the A/X-ratio, which is similar in rye and wheat [18,20], but higher in RSM compared to SBM [18]. Readily fermentable and more soluble AX are usually more substituted and characterized by a higher A/X-ratio [37]. However, solubility and fermentability of AX are reduced with increasing cross-linkages to lignin, ferulic acid or other polysaccharides and by a higher molecular weight of AX [18,38]. As discussed below, intestinal microbiota was shaped differently by the RSM-based diets in the present study compared to SBM. This might be related to the higher dietary and intestinal A/X-ratio of RSM. However, the high content of insoluble dietary fiber (IDF) and acid detergent lignin (ADL) in the RSM-based diets indicating a high degree of lignification might have reduced fermentability, as indicated by the reduced concentration of SCFA in RSM-fed pigs. Therefore, A/X-ratio could not be used as an indicator of degradability of AX in the current study.

### 4.2. Apparent Digestibility of NSP

The apparent colonic and total tract digestibility of total and soluble NSP in rye-fed pigs was higher compared to pigs receiving wheat-based diets. A higher ATTD of TDF and arabinose in rye-fed pigs was associated with an increased microbial fermentation in the large intestine [16]. In rye compared to wheat a higher fermentative activity might be related to the higher solubility of rye AX, the higher content of β-glucans and the higher total amount of dietary fiber [16,37]. Therefore, the digestibility of NSP in the current study might have been caused by a higher uptake of fermentable fiber with the rye-based diets, including more sNSP and soluble AX. Moreover, wheat AX might be less degradable, because of cross-linkages to other polysaccharides such as cellulose [37]. Cellulose is a major plant cell wall component, which is highly insoluble, poorly fermentable, and has a higher concentration in wheat than in rye [18]. The increased concentrations of digesta SCFA and the higher relative abundance of major plant cell wall-degrading bacterial species in the jejunum indicate an enhanced bacterial fiber degradation and can further explain the higher digestibility of NSP in rye-fed pigs of the present study.

With respect to NSP digestibility in RSM-fed pigs, values for colonic and total tract digestibility of total and iNSP was lower than in SBM-fed pigs. Similarly, ATTD of neutral detergent fiber (NDF), acid detergent fiber (ADF), carbohydrates, and dietary fiber was lower in other studies investigating the feeding of RSM instead of SBM to pigs [39,40]. However, one of these studies did not show a different ATTD of NSP and cellulose between RSM and SBM [39]. Microbial fiber fermentation and metabolic activity might be reduced as RSM is more lignified and insoluble than SBM [18]. This is reflected in the greater amount of IDF and iNSP in RSM-based diets of the current study and the lower SCFA concentration in jejunum, colon, and feces of RSM-fed pigs compared to SBM.

Surprisingly, the present values for colonic digestibility of NSP were higher than ATTD of NSP. In contrast, other studies show an increasing digestibility of NSP and AX in caudal direction [16,38]. However, the values of NSP digestibility were numerically in the same range, and furthermore, the determination of digestibility in feces is susceptible to interference. Bacteria may act as “non-dietary interfering substance” in NSP analytical procedures resulting in overestimated values of NSP concentration that are higher than the true diet derived NSP concentration [41]. Considering the higher number of bacteria in feces compared to colonic digesta and that in pigs and humans bacterial mass represents 40–50% of fecal DM [42,43], the interfering effect would be more pronounced with respect to ATTD of NSP compared to colonic digestibility. Additionally, some bacteria produce exopolysaccharides such as colonic acid (*Enterobacteria*) or dextran (*Lactobacillales*), which might have contributed to increased fecal concentration of NSP [44].

### 4.3. Bacterial Metabolites and Composition of the Microbiome

In the current study, compared to wheat, rye-fed pigs had a higher concentration of SCFA in the jejunum and colon. As shown in a study comparing the feeding of an AX-rich diet (65% rye-flakes) with a diet based on wheat flour, complex cereal polysaccharides such as AX are not degraded enzymatically in the small intestine and might therefore promote the growth of SCFA-producing bacteria in the distal parts of the intestine [38]. Another study focused on the feeding of wheat and rye breads to pigs, demonstrating that increased SCFA-production in the small intestine might be related to the more soluble fraction of dietary fiber, which is readily fermentable, whilst insoluble fractions would be degraded more distally [37]. Moreover, rye might cause a higher bacterial production of butyrate due to the structure of AX and the lower content of cellulose [45]. Therefore, the high concentration of TDF and SDF in the rye-based diets of this study may have provided more substrate for growth of SCFA-producing bacteria. Positive effects of SCFA derived from fermentation of dietary fiber on gut and animal health have been widely reviewed, including the use of acetate as an energy source and butyrate as the main fuel for colonocytes and its efficiency against possible pathogens [7,46]. In the present study, only data on digesta concentration of SCFA are available, but SCFA are absorbed rapidly via the gut wall [47]. Rye-derived SCFA might be of systemic use as peripheral blood concentration of SCFA was increased by an AX-rich diet based on rye-flakes compared to a wheat flour-based diet [48]. Despite the increased colonic concentration of SCFA and especially butyrate in the current study, no changes of the microbial community were determined in colonic digesta. This might be related to cross-feeding of AX-degrading bacteria, e.g., between *Bifidobacteria* and butyrate-producing bacteria, and to an increased abundance of the phosphotransferase system (kl02060) regulating carbohydrate uptake into bacterial cells [22]. Nevertheless, it is likely that rye compared to wheat enhanced the production of bacterial metabolites by providing a higher amount of easier fermentable substrate without a shift in the microbial community.

Compared to SBM-fed pigs, concentration of SCFA was lower in RSM-fed pigs in the jejunum and tendentially in the colon which indicates a lower metabolic activity of the resident microbiota. In contrast, previous studies showed an equal level of SCFA between RSM and SBM [24,25,28]. However, compared to these studies, the inclusion level of RSM and consequently the content of lignin and IDF was higher in the present study. A high degree of lignification may hamper enzymatic as well as microbial fiber degradation in RSM-fed pigs [39]. In combination with the lower digestibility of NSP and DM, this could explain the lower SCFA compared to SBM-fed pigs. Additionally, SCFA might have been absorbed rapidly or used by other microbiota in the sense of cross-feeding [49].

In accordance with a recent meta-analysis that identified *Firmicutes*, *Proteobacteria*, and *Bacteroidetes* as the most abundant phyla of the core microbiome in pigs [50], *Firmicutes* and *Bacteroidetes* were the predominant phyla of the pigs in the current study.

In rye-fed piglets of this study, composition of the microbial community was only different to wheat-fed pigs in the jejunum. This was unexpected, because according to other studies, the major impact of rye on the microbial community is expected in the proximal parts of the large intestine [16,23,38]. A higher relative abundance of *Firmicutes* in the small intestine might be explained by the higher content of sNSP in the rye-based diets, since more insoluble substrate would be degraded further distally [7]. The phylum *Firmicutes* contains many plant cell wall-degrading species including *Clostridium sensu stricto 1* and *Terrisporobacter*. These two genera are known to ferment complex indigestible plant polysaccharides such as hemicellulose and cellulose [51]. Therefore, the fiber provided by the rye-based diets might have served as a substrate and stimulated *Firmicutes*’ proliferation. *Firmicutes* contains many butyrate-producers [52], therefore an increased abundance could be considered as a positive effect of the feeding of rye. However, SCFA are absorbed primarily in the caecum and proximal colon, and might not utilized by the host in the same extent in the jejunum [47].

*Proteobacteria* had a lower jejunal relative abundance in rye-fed pigs of the current study. Many putative pathogens such as *E. coli* and *Salmonella* belong to *Proteobacteria* and a lower presence of *Proteobacteria* was associated with an increased intestinal barrier function and a higher anti-inflammatory capacity of the local immune system [53].

In contrast to an increased concentration of SCFA in jejunal digesta, the analysis of microbial diversity in rye-fed pigs showed a lower richness compared to wheat. It is possible that the increased production of metabolites might be driven by only a few selected genera of *Firmicutes* which were more abundant in the rye-fed pigs.

With respect to effects of RSM on the relative abundance of microbiota compared to SBM, the present study resulted in a reduced abundance of *Firmicutes* at the three sampling sites. As mentioned above, *Firmicutes* contains many fiber-degrading species and is known to produce SCFA. The lower NSP and DM digestibility together in RSM-fed pigs compared to SBM indicate that the fiber provided by the RSM-based diets was not used as a suitable substrate for fermentation by microbiota, especially fiber-degrading *Firmicutes.* Consequently, the metabolic activity and growth of microbiota was lower as illustrated by the lower concentration of SCFA and the lower relative abundance of *Firmicutes*. In line with this, compared to alfalfa meal, wheat bran and pure cellulose containing a higher amount of IDF also reduced the relative abundance of *Firmicutes* in large intestinal mucosa of suckling pigs [54]. However, another study investigating the feeding of RSM instead of SBM showed an increase of *Firmicutes*, although the lower inclusion level and lower content of IDF might have prevented an inverse result [55]. A higher ratio of *Firmicutes:Bacteroidetes* was associated with a reduction of the incidence of diarrhea and infections [55]. Nonetheless, previously published results of the present study showed that the fecal score of the pigs was in a physiological range throughout the trial [14]. Within *Firmicutes*, the predominant genera *Clostridium sensu stricto 1* and *Terrisporobacter* were also decreased by RSM in the current study. This might explain the reduced concentration of SCFA in RSM-fed pigs because another study showed a strong correlation between the relative abundance of these genera with the production of large amounts of metabolites from plant fiber [51].

In line with another study investigating the feeding of RSM [25], RSM increased the relative abundance of *Actinobacteria* along the gastro-intestinal tract in the current study. In contrast, another study did not show differing values of abundance of *Actinobacteria* in RSM- or SBM-fed pigs [55]. *Actinobacteria* efficiently use hemicellulose and cellulose [52], both mainly insoluble fiber fractions. Despite RSM and SBM containing similar amounts of cellulose [18] the higher inclusion of RSM compared to SBM and the higher content of IDF in RSM-diets might have promoted growth of *Actinobacteria* in RSM-fed pigs. In the jejunum, *Bifidobacterium*, a genus belonging to *Actinobacteria*, was also increased by the feeding of RSM. *Bifidobacterium* is considered to improve gut health [7,56] and was increased by the feeding of cellulose in another study [57].

As mentioned above, an increased relative abundance of *Proteobacteria* may be a risk for animal health. Nevertheless, the pigs of both RSM and SBM groups were in a good condition throughout the trial. *Proteobacteria* contains many proteolytic genera [58]. RSM may lead to a lower AID of protein and amino acids than SBM [59] which also was demonstrated in this study [14]. However, there was no correlation detected between AID of crude protein and total amino acids and the jejunal relative abundance of *Proteobacteria*. It is likely that the protein was not available as a substrate for growth of *Proteobacteria*, because IDF in RSM caused an encapsulation in the rigid cell wall and an increased digesta passage rate [39].

In RSM-fed pigs, the increased relative abundance of *Bacteroidetes* is most likely related to the higher content of IDF than in SBM-based diets. *Bacteroidetes* as the second most abundant phylum of the gut microbiota in pigs [50] was also increased by resistant starch in humans [60] and by corn bran in pigs [61], both sources of IDF. In contrast, a study investigating the feeding of RSM instead of SBM to pigs showed a lower abundance of *Bacteroidetes* which was related to the high pectin content [25]. An increased abundance of *Bacteroidetes* is associated with weight loss in humans, mice [62], and pigs [63]. The negative correlation between abundance of *Bacteroidetes* and ADG in the last six days of the trial might suggest that the shift towards more colonic *Bacteroidetes* was connected to the reduced weight gain in RSM-fed pigs.

## 5. Conclusions

In conclusion, compared to wheat, the higher amount and solubility of NSP from rye resulted in an increased degradation and fermentation of NSP and in a higher metabolic activity of intestinal microbiota. However, relative abundance of large intestinal microbiota was not different between pigs fed rye and wheat. RSM caused a lower bacterial metabolic activity than SBM. The higher fiber content of RSM-based diets was expected to increase fermentation, but 30% inclusion of RSM might have provided an excess of IDF. Still, RSM lead to a lower abundance of common fiber degraders of the predominant phylum *Firmicutes* and an increased abundance of IDF degrading *Actinobacteria* and *Bacteroidetes*. Further research is needed to better understand host–microbiota interaction and to improve feeding concepts with a targeted use of dietary fiber.

## Figures and Tables

**Table 1:** Analyzed content of DM and dietary fiber of the experimental diets.

Item, g/kg (as-Fed Basis)	Diet			
	W-SBM	W-RSM	R-SBM	R-RSM
DM	931	937	931	928
NDF	154	153	163	141
ADF	26.3	74.1	23.0	70.5
ADL	2.2	28.2	4.3	24.9
NSP				
Total	89.6 (12.8) ^1^	102 (14.2)	104 (24.2)	128 (34.6)
Fucose	0.86 (0.03)	0.82 (0.02)	0.99 (0.01)	0.91 (0.08)
Rhamnose	0.79 (0.07)	0.81 (0.09)	0.76 (0.11)	1.00 (0.13)
Arabinose	18.2 (2.02)	25.1 (2.82)	21.3 (4.46)	30.5 (7.67)
Galactose	13.1 (2.13)	8.20 (1.82)	14.1 (2.38)	7.72 (0.90)
Glucose	27.0 (0.86)	38.0 (2.50)	30.7 (5.35)	48.2 (11.8)
Mannose	3.43 (2.17)	2.83 (1.90)	4.81 (4.03)	5.21 (3.22)
Xylose	24.2 (5.18)	23.9 (4.92)	27.5 (7.13)	30.6 (10.8)
Galacturonic acid	1.90 (0.26)	1.71 (0.08)	4.00 (0.65)	3.39 (0.08)
Glucuronic acid	0.18 (0.06)	0.18 (0.05)	0.20 (0.05)	0.14 (0.03)
Total AX ^2^	42.4 (7.2)	49.0 (7.7)	48.9 (11.6)	61.1 (18.5)
Ratio A/X ^3^	0.75	1.05	0.77	1.00
TDF	135	164	152	188
SDF ^4^	27.6	27.1	37.1	42.4
IDF	108	137	115	146

W-SBM, wheat/soybean meal; W-RSM, wheat/rapeseed meal; R-SBM, rye/soybean meal; R-RSM, rye/rapeseed meal; DM, dry matter; NDF, neutral detergent fiber; ADF, acid detergent fiber; ADL, acid detergent lignin; NSP, non-starch-polysaccharides; AX, arabinoxylans; TDF: total dietary fiber; SDF, soluble dietary fiber; IDF, insoluble dietary fiber. ^1^ Values in parentheses are concentration of soluble fraction: total concentration of each component–concentration of the respective insoluble fraction. ^2^ Arabinose + xylose. ^3^ A/X: Arabinose/Xylose. ^4^ SDF, soluble dietary fiber: TDF–IDF.

**Table 2:** Effects of the experimental diets on apparent digestibility of DM and NSP in weaned piglets ^1^.

Digestibility, %	Diet					*p*-Value		
	W-SBM	W-RSM	R-SBM	R-RSM	SEM	CER	PM	CER * PM
Jejunum								
DM	73.3	53.1	70.6	57.4	1.9	0.787	<0.001	0.246
Colon ascendens								
DM	86.7	80.0	85.4	78.4	0.8	0.224	<0.001	0.925
NSP								
Total	70.4	64.5	77.4	71.1	1.0	<0.001	<0.001	0.903
Fucose	81.8	67.8	79.0	64.4	1.7	0.241	<0.001	0.890
Rhamnose	55.0	26.5	53.9	37.8	4.7	0.573	0.018	0.495
Arabinose	69.5	72.9	74.2	75.3	0.7	0.011	0.097	0.375
Galactose	92.0	70.1	89.5	64.1	2.1	0.043	<0.001	0.394
Glucose	62.0	61.3	75.3	72.1	1.2	<0.001	0.222	0.448
Mannose	74.8 ^a^	48.8 ^b^	78.7 ^a^	67.6 ^a^	2.3	0.001	<0.001	0.020
Xylose	70.0	71.1	76.9	76.7	0.9	<0.001	0.757	0.639
Galacturonic acid	83.1 ^a^	−29.7 ^c^	91.7 ^a^	31.3 ^b^	7.6	<0.001	<0.001	<0.001
Glucuronic acid	47.3 ^b^	40.6 ^b^	72.3 ^a^	42.0 ^b^	2.6	0.001	<0.001	0.002
Soluble NSP ^2^	70.7	71.4	86.3	88.2	1.8	<0.001	0.624	0.821
Insoluble NSP	70.4	63.4	74.8	64.7	1.1	0.116	<0.001	0.405
Total AX	69.8	72.1	75.7	76.0	0.8	0.001	0.370	0.467
Soluble AX ^3^	81.9	88.3	94.5	96.2	1.5	<0.001	0.130	0.362
Insoluble AX	67.3	69.0	69.9	67.2	0.8	0.807	0.783	0.189
Ratio A/X ^4^	0.82	1.05	0.94	1.14	0.02	<0.001	<0.001	0.640
Feces ^5^								
DM	87.7	80.7	87.1	77.8	0.7	0.035	<0.001	0.160
NSP								
Total	65.3	59.5	74.8	62.5	1.2	0.001	<0.001	0.073
Fucose	80.9	64.0	74.0	56.7	1.9	0.011	<0.001	0.948
Rhamnose	41.5	1.0	25.4	0.5	3.7	0.129	<0.001	0.155
Arabinose	60.5 ^b^	68.1 ^a^	67.7 ^a^	67.1 ^a,b^	1.0	0.096	0.058	0.027
Galactose	90.6 ^a^	67.6 ^b^	87.4 ^a^	51.3 ^c^	2.5	<0.001	<0.001	<0.001
Glucose	51.1 ^b^	51.2 ^b^	71.4 ^a^	59.4 ^b^	1.7	<0.001	0.018	0.017
Mannose	83.7 ^a^	57.4 ^c^	87.1 ^a^	74.3 ^b^	2.0	<0.001	<0.001	0.002
Xylose	66.7	69.2	73.4	70.3	1.0	0.040	0.865	0.131
Galacturonic acid	85.5 ^a^	−45.2 ^c^	93.4 ^a^	11.7 ^b^	9.1	<0.001	<0.001	<0.001
Glucuronic acid	30.8 ^b^	29.9 ^b^	66.3 ^a^	28.5 ^b^	2.9	<0.001	<0.001	<0.001
Soluble	71.4	54.9	85.8	79.7	2.5	<0.001	0.004	0.162
Insoluble	64.1 ^b^	59.9 ^bc^	71.2 ^a^	55.7 ^c^	1.2	0.413	<0.001	0.003
Total AX	64.0	68.7	70.9	68.7	0.9	0.060	0.508	0.061
Soluble AX	81.5	81.9	90.9	93.2	1.8	0.005	0.686	0.779
Insoluble AX	60.4 ^a,b^	66.2 ^a^	64.7 ^a,b^	58.0 ^b^	1.0	0.282	0.788	0.001
Ratio A/X	0.95	1.18	1.01	1.19	0.02	0.143	<0.001	0.367

W-SBM, wheat/soybean meal; W-RSM, wheat/rapeseed meal; R-SBM, rye/soybean meal; R-RSM, rye/rapeseed meal; SEM, standard error of the mean; CER, cereal; PM, protein meal; DM, dry matter; NSP, non-starch-polysaccharides; AX, arabinoxylans. ^1^ Data are presented as means (*n* = 11); p-values indicate effects of the factors cereal (CER), protein meal (PM) and their interaction (CER * PM). ^2^ Soluble NSP: total NSP–insoluble NSP. ^3^ Soluble AX, total AX–insoluble AX. ^4^ A/X, ratio of concentration of arabinose and xylose in digesta or feces. ^5^ R-SBM: *n* = 10 (lack of collectable feces). ^a, b, c^ Values within a row with different superscripts differ significantly at *p* ≤ 0.05 (Tukey test).

**Table 3:** Effects of the experimental diets on *N*-acetylneuraminic acid concentration in ileal digesta ^1^.

Item	Diet					*p*-Value		
	W-SBM	W-RSM	R-SBM	R-RSM	SEM	CER	PM	CER * PM
Neu5Ac, g/kg DM	1.15	1.41	1.25	1.47	0.11	0.702	0.294	0.932
Ratio Neu5Ac/TiO2	11.7	21.3	14.1	21.6	2.3	0.761	0.070	0.813

W-SBM, wheat/soybean meal; W-RSM, wheat/rapeseed meal; R-SBM, rye/soybean meal; R-RSM, rye/rapeseed meal; SEM, standard error of the mean; CER, cereal; PM, protein meal; Neu5Ac, *N*-acetylneuraminic acid; DM, dry matter. ^1^ Data are presented as means (W-SBM, W-RSM, R-SBM: *n* = 7; R-RSM: *n* = 6, lack of collectable digesta); *p*-values indicate effects of the factors cereal (CER), protein meal (PM) and their interaction (CER * PM).

**Table 4:** Effects of the experimental diets on bacterial metabolites in digesta and feces of weaned piglets ^1^.

Concentration, µmol/g DM	Diet					*p*-Value		
	W-SBM	W-RSM	R-SBM	R-RSM	SEM	CER	PM	CER * PM
Jejunum								
SCFA	89.6	30.8	192	104	13.6	<0.001	0.001	0.511
Acetic acid	79.0	27.8	172	96.1	12.1	<0.001	0.002	0.517
Propionic acid	0.47	0.68	3.60	1.26	0.43	0.024	0.188	0.115
i-butyric acid	1.33	0.94	1.42	1.33	0.11	0.288	0.286	0.509
n-butyric acid	8.43	1.12	14.0	5.51	1.58	0.097	0.010	0.845
i-valeric acid	0.08	0.00	0.27	0.03	0.05	0.195	0.072	0.353
n-valeric acid	0.24	0.28	0.36	0.28	0.03	0.351	0.709	0.342
Ammonia	37.9	29.7	53.7	37.8	3.4	0.073	0.070	0.551
l-lactate	177	213	303	133	32	0.717	0.295	0.112
d-lactate	5.64	9.83	8.92	7.15	2.04	0.944	0.775	0.483
Colon ascendens								
SCFA	990	922	1316	1040	48	0.016	0.057	0.244
Acetic acid	536	489	743	566	30	0.014	0.049	0.243
Propionic acid	251	236	314	280	12	0.034	0.323	0.706
i-butyric acid	11.0	9.56	14.2	9.78	0.48	0.036	0.001	0.064
n-butyric acid	153	141	191	143	7	0.172	0.041	0.197
i-valeric acid	10.9	8.58	13.9	7.61	0.74	0.430	0.002	0.133
n-valeric acid	28.9	37.0	39.9	33.7	2.3	0.402	0.836	0.124
Ammonia	51.5	52.9	65.5	41.5	3.6	0.849	0.116	0.078
l-lactate	22.4	34.1	40.8	28.4	3.9	0.425	0.965	0.133
d-lactate	7.69	11.0	9.17	7.80	1.31	0.754	0.723	0.390
Feces ^2^								
SCFA	592	529	688	571	34	0.308	0.187	0.694
Acetic acid	283	257	358	291	17	0.112	0.171	0.537
Propionic acid	154	133	163	140	9	0.642	0.236	0.972
i-butyric acid	15.1	15.9	21.6	15.6	1.0	0.106	0.168	0.079
n-butyric acid	97.1	80.8	93.2	81.3	6.5	0.898	0.296	0.870
i-valeric acid	21.0	21.5	31.0	21.5	1.4	0.065	0.094	0.063
n-valeric acid	22.7	20.8	21.9	22.0	1.3	0.943	0.755	0.711
Ammonia	78.4	84.5	86.0	78.1	5.6	0.958	0.939	0.552
l-lactate	12.0	19.2	12.9	15.5	2.0	0.714	0.223	0.562
d-lactate	6.42	11.7	6.45	10.0	1.45	0.778	0.135	0.770

DM, dry matter; W-SBM, wheat/soybean meal; W-RSM, wheat/rapeseed meal; R-SBM, rye/soybean meal; R-RSM, rye/rapeseed meal; SEM, standard error of the mean; CER, cereal; PM, protein meal; SCFA, short-chain fatty acids; ^1^ Data are presented as means (*n* = 11); *p*-values indicate effects of the factors cereal (CER), protein meal (PM), and their interaction (CER * PM). ^2^ R-SBM: *n* = 10 (lack of collectable feces).

**Table 5:** Effects of the experimental diets on ecological indices of the intestinal microbiota of weaned piglets ^1^.

	Diet					*p*-Value		
	W-SBM	W-RSM	R-SBM	R-RSM	SEM	CER	PM	CER * PM
Jejunum ^2^								
Richness	42.3	72.8	37.2	38.9	5.1	0.047	0.098	0.138
Shannon Index	1.53	1.94	1.61	1.65	0.08	0.522	0.165	0.247
Evenness	0.420	0.475	0.448	0.462	0.011	0.781	0.223	0.475
Colon ascendens								
Richness	186	202	203	190	6	0.811	0.899	0.246
Shannon Index	3.46	3.65	3.69	3.88	0.06	0.055	0.112	0.957
Evenness	0.666	0.688	0.699	0.743	0.010	0.060	0.149	0.633
Feces								
Richness	159	177	192	182	6	0.163	0.748	0.286
Shannon Index	3.20	3.38	3.35	3.74	0.09	0.176	0.128	0.558
Evenness	0.633	0.652	0.639	0.720	0.023	0.213	0.096	0.300

W-SBM, wheat/soybean meal; W-RSM, wheat/rapeseed meal; R-SBM, rye/soybean meal; R-RSM, rye/rapeseed meal; SEM, standard error of the mean; CER, cereal; PM, protein meal. ^1^ Data are presented as means (*n* = 11); p-values indicate effects of the factors cereal (CER), protein meal (PM), and their interaction (CER * PM). ^2^ W-RSM: *n* = 10 (DNA-extract not amplifiable).

**Table 6:** Effects of the experimental diets on the relative abundance (%) of bacterial phyla ^1^.

	Diet					*p*-Value		
	W-SBM	W-RSM	R-SBM	R-RSM	SEM	CER	PM	CER * PM
Jejunum ^2^								
Actinobacteria	0.550	5.40	1.36	2.72	0.673	0.467	0.019	0.177
Bacteroidetes	0.064	3.11	0.089	0.174	0.686	0.292	0.258	0.284
Firmicutes	98.4 ^a^	88.7 ^b^	98.2 ^a^	96.7 ^a^	1.1	0.039	0.004	0.032
Proteobacteria	0.749	2.60	0.131	0.407	0.255	0.002	0.019	0.077
Colon ascendens								
Actinobacteria	0.344	2.61	1.44	3.00	0.435	0.380	0.029	0.678
Bacteroidetes	17.5	23.4	18.2	26.1	1.4	0.540	0.014	0.713
Firmicutes	80.0	72.3	79.1	68.2	1.7	0.423	0.005	0.607
Proteobacteria	1.82	1.00	0.702	2.10	0.358	0.989	0.688	0.131
Spirochaetes	0.202	0.082	0.350	0.133	0.044	0.245	0.053	0.569
Feces								
Actinobacteria	0.749	3.61	2.08	4.86	0.516	0.182	0.005	0.965
Bacteroidetes	10.6	12.3	11.0	16.3	1.1	0.322	0.123	0.415
Firmicutes	86.7	82.4	85.6	76.9	1.3	0.184	0.012	0.377
Proteobacteria	0.961	1.19	0.247	1.35	0.221	0.533	0.137	0.323
Spirochaetes	0.671	0.098	0.387	0.179	0.145	0.730	0.189	0.537
Tenericutes	0.061	0.056	0.049	0.058	0.012	0.844	0.956	0.783

W-SBM, wheat/soybean meal; W-RSM, wheat/rapeseed meal; R-SBM, rye/soybean meal; R-RSM, rye/rapeseed meal; SEM, standard error of the mean; CER, cereal; PM, protein meal. ^1^ Data are presented as means (*n* = 11); *p*-values indicate effects of the factors cereal (CER), protein meal (PM) and their interaction (CER * PM). ^2^ W-RSM: *n* = 10 (DNA-extract not amplifiable). ^a, b^ Values within a row with different superscripts differ significantly at *p* ≤ 0.05 (Tukey test).

## Data Availability

Data supporting reported results is contained within the article and the Supplementary Materials.

## Supplementary Materials

The following are available online at https://www.mdpi.com/article/10.3390/ani12010109/s1, Table S1: Effects of the experimental diets on the relative abundance (%) of bacterial orders, Table S2: Effects of the experimental diets on the relative abundance (%) of bacterial genera.
