# Supplementary material for: Effects of Dietary Cereal and Protein Source on Fiber Digestibility, Composition, and Metabolic Activity of the Intestinal Microbiota in Weaner Piglets
Source: Animals (Basel). 2022 Jan 4;12(1):109. doi: 10.3390/ani12010109 (PMC8749901; doi:10.3390/ani12010109)
Supplement: Supplementary file 1 [file animals-12-00109-s001.zip › animals-1530146-supplementary.pdf]

Table S1. Effects of the experimental diets on the relative abundance (%) of bacterial orders[1]

	Diet					*P*-Value		
	W-SBM	W-RSM	R-SBM	R-RSM	SEM	*CER*	*PM*	*CER*PM*
Jejunum[2]								
Clostridiales	55.5	16.0	71.4	51.9	4.8	0.002	0.001	0.208
Lactobacillales	34.9	70.3	25.3	43.1	4.5	0.024	0.002	0.266
Erysipelotrichales	5.30	1.66	1.40	1.61	0.60	0.086	0.135	0.094
Coriobacteriales	0.158	0.432	0.025	0.050	0.059	0.024	0.179	0.263
Enterobacteriales	0.129	1.30	0.004	0.249	0.181	0.089	0.043	0.176
Bifidobacteriales	0.057	3.24	1.45	2.65	0.593	0.733	0.069	0.402
Colon ascendens								
Clostridiales	44.3	26.0	54.0	38.4	2.6	0.016	< 0.001	0.751
Lactobacillales	32.4	43.1	22.6	22.8	2.8	0.005	0.292	0.309
Bacteroidales	17.5	23.4	18.2	26.1	1.4	0.540	0.014	0.713
Selenomonadales	2.37	2.51	1.60	4.09	0.42	0.626	0.121	0.163
Aeromonadales	1.67	0.610	0.318	1.37	0.328	0.657	0.997	0.115
Erysipelotrichales	0.920	0.764	0.916	2.881	0.325	0.094	0.150	0.092
Coriobacteriales	0.260	1.23	0.257	2.18	0.317	0.442	0.022	0.439
Spirochaetales	0.200	0.082	0.346	0.133	0.043	0.245	0.055	0.573
Bifidobacteriales	0.084	1.37	1.19	0.820	0.331	0.683	0.494	0.223
Gastranaerophilales	0.056	0.486	0.077	0.303	0.065	0.517	0.011	0.414
Desulfovibrionales	0.028	0.081	0.030	0.456	0.100	0.352	0.238	0.356
Faeces								
Clostridiales	50.3	32.9	59.9	39.5	2.7	0.088	< 0.001	0.751
Lactobacillales	32.8	46.7	23.1	28.6	3.3	0.032	0.127	0.509
Bacteroidales	10.6	12.3	11.0	16.3	1.1	0.322	0.123	0.415
Selenomonadales	2.58	2.02	1.56	5.68	0.60	0.252	0.125	0.046
Erysipelotrichales	1.02	0.897	1.04	3.03	0.287	0.047	0.083	0.052
Aeromonadales	0.866	0.371	0.051	0.908	0.220	0.755	0.684	0.134
Spirochaetales	0.671	0.098	0.387	0.179	0.145	0.730	0.189	0.537
Coriobacteriales	0.624	1.77	0.488	3.39	0.357	0.255	0.003	0.179
Bifidobacteriales	0.125	1.83	1.59	1.47	0.415	0.514	0.349	0.279
Desulfovibrionales	0.079	0.186	0.052	0.263	0.024	0.543	< 0.001	0.216
Mollicutes RF39	0.061	0.056	0.049	0.058	0.012	0.844	0.956	0.783
unknown *WPS-2*	0.055	0.197	0.461	0.227	0.084	0.202	0.785	0.272

W-SBM, wheat/soybean meal; *W-RSM*, wheat/rapeseed meal; *R-SBM*, rye/soybean meal; *R-RSM*, rye/rapeseed meal; *SEM*, standard error of the mean; *CER*, cereal; *PM*, protein meal

[1] Data are presented as means (*n*=11); *P*-values indicate effects of the factors cereal (*CER*), protein meal (*PM*) and their interaction (*CER*PM*)

[2] W-RSM: *n* = 10 (DNA-extract not amplifiable)

Table S2. Effects of the experimental diets on the relative abundance (%) of bacterial genera[1]

	Diet					*P*-Value		
	W-SBM	W-RSM	R-SBM	R-RSM	SEM	*CER*	*PM*	*CER*PM*
Jejunum[2]								
Clostridium sensu stricto 1	44.3[a]	3.69[b]	47.5[a]	36.4[a]	3.94	0.005	< 0.001	0.021
Streptococcus	22.8	37.8	22.0	32.5	3.8	0.692	0.101	0.774
Lactobacillus	11.2	31.4	3.01	10.1	3.9	0.050	0.069	0.376
Terrisporobacter	8.00	2.21	15.4	8.73	1.53	0.018	0.033	0.876
Turicibacter	5.23	1.34	1.32	1.56	0.58	0.094	0.099	0.062
Romboutsia	1.81	0.828	1.53	1.52	0.349	0.774	0.493	0.497
Sarcina	0.354	6.74	6.59	4.66	1.828	0.577	0.551	0.269
unknown *Streptococcaceae*	0.284[a]	0.049[b]	0.214[ab]	0.209[ab]	0.027	0.360	0.017	0.022
unknown.*Lactobacillales*	0.118	0.037	0.057	0.168	0.030	0.559	0.799	0.111
Intestinibacter	0.113	0.050	0.059	0.056	0.018	0.504	0.357	0.408
Bifidobacterium	0.047	3.20	1.32	2.64	0.555	0.744	0.046	0.404
Olsenella	0.039	0.159	0.015	0.016	0.021	0.037	0.125	0.130
unknown *Peptostreptococcaceae*	0.035	0.029	0.058	0.072	0.009	0.080	0.805	0.590
Colon ascendens								
Clostridium sensu stricto 1	16.0	1.47	20.2	7.33	1.77	0.085	< 0.001	0.775
Agathobacter	4.20	3.32	4.00	3.38	0.33	0.918	0.273	0.844
Blautia	2.42	2.97	2.71	3.59	0.21	0.280	0.096	0.698
Coprococcus 3	0.778	1.14	1.13	1.21	0.088	0.223	0.214	0.421
Alloprevotella	0.766	0.688	1.05	0.780	0.128	0.481	0.516	0.722
Dorea	0.546	0.891	0.909	1.18	0.116	0.159	0.184	0.879
Intestinibacter	0.484[a]	0.131[b]	0.269[ab]	0.306[ab]	0.045	0.820	0.069	0.026
Lachnospira	0.455	0.633	0.438	0.698	0.059	0.839	0.071	0.730
Lachnospiraceae NC2004 group	0.350	0.489	0.614	0.362	0.057	0.551	0.622	0.093
Faecalibacterium	0.341	0.793	0.725	2.35	0.311	0.113	0.090	0.333
Coprococcus 1	0.260	0.381	0.308	0.164	0.074	0.578	0.940	0.384
Fusicatenibacter	0.226	0.158	0.154	0.354	0.035	0.366	0.342	0.056
Lachnoclostridium	0.197	0.199	0.140	0.161	0.027	0.403	0.838	0.863
Christensenellaceae R-7 group	0.178	0.061	0.390	0.239	0.071	0.181	0.354	0.908
Anaerovibrio	0.173	0.104	0.237	0.190	0.036	0.319	0.442	0.882
Lachnospiraceae FCS020 group	0.152	0.103	0.073	0.099	0.012	0.069	0.604	0.103
Acidaminococcus	0.142	0.131	0.138	0.105	0.026	0.778	0.678	0.844
Asteroleplasma	0.140	0.245	0.062	0.380	0.067	0.831	0.120	0.428
Catenisphaera	0.136	0.032	0.157	0.345	0.048	0.082	0.657	0.125
Catenibacterium	0.107	0.137	0.133	1.31	0.293	0.313	0.311	0.335
Coprococcus 2	0.097	0.170	0.051	0.067	0.026	0.151	0.389	0.577
Family XIII AD3011 group	0.095	0.045	0.148	0.070	0.013	0.128	0.014	0.594
Bifidobacterium	0.084	1.37	1.19	0.820	0.331	0.683	0.494	0.223
Collinsella	0.071	0.152	0.120	0.418	0.053	0.129	0.069	0.292
Holdemanella	0.067	0.172	0.219	0.392	0.041	0.019	0.075	0.659
Butyricicoccus	0.066	0.033	0.021	0.094	0.011	0.695	0.327	0.013
Acetitomaculum	0.056	0.096	0.145	0.072	0.029	0.583	0.779	0.338
Fournierella	0.045	0.073	0.165	0.106	0.019	0.047	0.675	0.252
Desulfovibrio	0.028	0.081	0.030	0.456	0.100	0.352	0.238	0.356
Intestinimonas	0.020	0.059	0.029	0.046	0.008	0.897	0.078	0.477
Enterorhabdus	0.018	0.040	0.014	0.042	0.006	0.954	0.023	0.779
Faeces								
Streptococcus	26.1	32.7	16.2	18.6	3.1	0.057	0.468	0.729
Clostridium sensu stricto 1	20.3	3.03	27.2	9.39	2.18	0.060	< 0.001	0.944
Lactobacillus	6.54	13.9	6.73	10.0	2.01	0.653	0.199	0.621

unknown.*Muribaculacea*	5.70	2.64	4.78	4.79	0.62	0.622	0.222	0.219
Terrisporobacter	3.08	0.883	7.77	3.03	0.669	0.004	0.004	0.268
Subdoligranulum	2.73	1.35	1.65	2.01	0.54	0.852	0.646	0.433
Agathobacter	2.15	2.27	1.21	1.30	0.29	0.104	0.861	0.973
Ruminococcaceae UCG-005	2.14	1.56	1.91	1.22	0.20	0.457	0.110	0.888
Prevotella 9	2.11	5.22	2.64	7.13	0.83	0.449	0.022	0.666
unknown *Lachnospiraceae*	1.81	2.22	1.62	2.66	0.20	0.757	0.076	0.430
unknown *Ruminococcaceae*	1.71	0.88	1.50	1.09	0.15	0.999	0.042	0.483
Blautia	1.71	2.83	1.78	2.80	0.26	0.974	0.039	0.922
Megasphaera	1.58	0.668	0.643	1.54	0.299	0.955	0.988	0.141
Christensenellaceae R-7 group	1.50	0.450	0.974	0.441	0.184	0.453	0.031	0.468
Ruminococcaceae UCG-002	1.26	1.47	0.913	1.34	0.197	0.558	0.438	0.783
Syntrophococcus	1.15	2.57	0.268	1.34	0.286	0.054	0.024	0.742
Prevotellaceae NK3B31 group	1.14	1.24	1.27	0.538	0.211	0.499	0.464	0.338
Ruminococcaceae NK4A214 group	0.817	1.09	0.974	1.05	0.134	0.831	0.532	0.725
Ruminococcus 2	0.770	0.433	0.317	0.265	0.093	0.098	0.295	0.441
Marvinbryantia	0.766	0.641	0.410	0.586	0.049	0.035	0.787	0.116
Roseburia	0.761	0.734	0.549	0.413	0.123	0.294	0.746	0.829
Treponema 2	0.671	0.098	0.387	0.177	0.145	0.729	0.188	0.538
Phascolarctobacterium	0.581	0.484	0.729	1.10	0.109	0.079	0.519	0.274
Ruminococcaceae UCG-008	0.532	0.153	1.11	0.377	0.077	0.001	< 0.001	0.118
Coprococcus 3	0.525	1.36	0.924	1.59	0.130	0.188	0.003	0.726
Ruminococcus 1	0.494	0.366	0.356	0.283	0.045	0.232	0.274	0.764
Dorea	0.483	0.845	0.637	1.20	0.141	0.367	0.106	0.721
Family XIII AD3011 group	0.468	0.187	0.534	0.289	0.049	0.368	0.007	0.841
Turicibacter	0.441	0.144	0.290	0.133	0.059	0.483	0.054	0.544
Prevotella 7	0.436	0.881	0.135	1.41	0.192	0.758	0.025	0.266
uminococcaceae UCG-014	0.422	0.533	0.554	0.603	0.053	0.356	0.464	0.772
Succinivibrio	0.393	0.179	0.051	0.832	0.181	0.670	0.439	0.178
Oribacterium	0.373	0.690	0.238	0.435	0.060	0.091	0.028	0.597
Lachnospiraceae NK4A136 group	0.332	0.220	0.397	0.231	0.053	0.725	0.203	0.805
Intestinibacter	0.308	0.098	0.264	0.312	0.035	0.206	0.228	0.059
unknown *(Family.Prevotellaceae)*	0.298	0.391	0.230	0.486	0.059	0.912	0.149	0.495
Lachnospiraceae NC2004 group	0.289	0.335	0.424	0.223	0.042	0.894	0.359	0.147
Olsenella	0.271	0.950	0.148	2.33	0.312	0.291	0.019	0.208
Rikenellaceae RC9 gut group	0.252	0.506	0.438	0.414	0.075	0.759	0.455	0.367
Collinsella	0.226	0.223	0.175	0.423	0.039	0.323	0.110	0.103
Prevotella 2	0.213	0.244	0.226	0.514	0.056	0.198	0.150	0.242
Lachnospiraceae NK3A20 group	0.191	0.249	0.103	0.270	0.050	0.742	0.278	0.598
Coprococcus 1	0.189	0.428	0.231	0.190	0.082	0.560	0.554	0.406
Catenisphaera	0.184	0.048	0.214	0.747	0.092	0.038	0.249	0.056
Lachnospira	0.178	0.354	0.175	0.216	0.048	0.468	0.265	0.488
Mogibacterium	0.172	0.137	0.081	0.131	0.018	0.189	0.840	0.247
Fusicatenibacter	0.148	0.160	0.101	0.366	0.041	0.323	0.087	0.117
Oscillospira	0.147	0.260	0.190	0.149	0.030	0.576	0.551	0.205
Catenibacterium	0.145	0.177	0.167	1.15	0.224	0.272	0.263	0.294
Lachnospiraceae ND3007 group	0.142	0.326	0.283	0.277	0.041	0.577	0.286	0.252
Prevotella 1	0.139	0.579	0.180	0.319	0.096	0.570	0.137	0.435
Solobacterium	0.130	0.082	0.078	0.194	0.025	0.548	0.486	0.099
unknown *Family XIII*	0.128	0.196	0.068	0.129	0.045	0.494	0.486	0.967
Bifidobacterium	0.125	1.83	1.59	1.47	0.415	0.514	0.349	0.279
Faecalibacterium	0.121	0.535	0.516	0.815	0.088	0.048	0.037	0.731
unknown *Veillonellaceae*	0.111	0.076	0.025	0.092	0.017	0.288	0.636	0.129
Lachnospiraceae FCS020 group	0.109	0.117	0.096	0.155	0.016	0.694	0.312	0.438

unknown *Streptococcaceae*	0.101	0.052	0.098	0.063	0.013	0.884	0.118	0.796
Intestinimonas	0.096	0.120	0.043	0.075	0.019	0.211	0.468	0.914
Ruminococcaceae UCG-010	0.088	0.125	0.076	0.094	0.022	0.643	0.557	0.842
Ruminococcaceae UCG-013	0.084	0.021	0.077	0.069	0.016	0.520	0.266	0.389
Desulfovibrio	0.079	0.186	0.052	0.263	0.024	0.543	< 0.001	0.216
Prevotellaceae UCG-003	0.078	0.033	0.176	0.097	0.018	0.022	0.075	0.622
Ruminococcaceae UCG-004	0.076	0.050	0.152	0.077	0.016	0.110	0.116	0.440
Lachnoclostridium	0.069	0.181	0.065	0.091	0.018	0.170	0.051	0.217
Holdemanella	0.069	0.236	0.227	0.314	0.039	0.128	0.102	0.598
Shuttleworthia	0.067	0.792	0.334	0.045	0.151	0.427	0.471	0.098
unknown *Mollicutes RF39*	0.061	0.056	0.049	0.058	0.012	0.844	0.956	0.783
unknown *WPS-2*	0.055	0.197	0.461	0.227	0.084	0.202	0.785	0.272
Ruminiclostridium 9	0.054	0.072	0.056	0.058	0.010	0.745	0.626	0.697
Alloprevotella	0.049	0.250	0.352	0.153	0.045	0.246	0.994	0.027
Ruminiclostridium 5	0.048	0.039	0.076	0.054	0.008	0.167	0.304	0.682
unknown *Atopobiaceae*	0.044	0.361	0.055	0.345	0.035	0.959	< 0.001	0.796
Fournierella	0.043	0.061	0.105	0.088	0.016	0.164	0.986	0.582
Sarcina	0.027	0.090	1.35	0.438	0.158	0.005	0.137	0.089
Candidatus_Soleaferrea	0.027	0.051	0.054	0.085	0.012	0.199	0.233	0.885
unknown *Bacteroidales*	0.024	0.107	0.077	0.145	0.024	0.345	0.120	0.879
Acetitomaculum	0.022	0.152	0.017	0.105	0.023	0.555	0.018	0.643
unknown *Erysipelotrichaceae*	0.021	0.061	0.034	0.006	0.011	0.353	0.779	0.132
Enterorhabdus	0.017	0.093	0.050	0.123	0.012	0.151	0.001	0.936
unknown *Coriobacteriales*	0.017	0.089	0.017	0.076	0.011	0.768	0.004	0.750

W-SBM, wheat/soybean meal; *W-RSM*, wheat/rapeseed meal; *R-SBM*, rye/soybean meal; *R-RSM*, rye/rapeseed meal; *SEM*, standard error of the mean; *CER*, cereal; *PM*, protein meal

[1] Data are presented as means (*n*=11); *P*-values indicate effects of the factors cereal (*CER*), protein meal (*PM*) and their interaction (*CER*PM*)

[2] W-RSM: *n* = 10 (DNA-extract not amplifiable)

[a, b] Values within a row with different superscripts differ significantly at $P \leq 0.05$ (Tukey Test)
